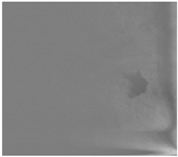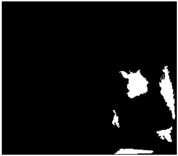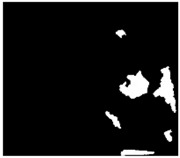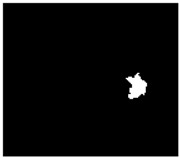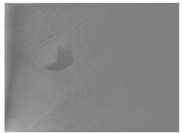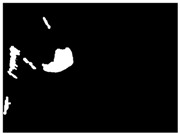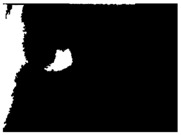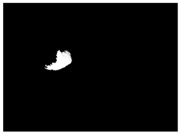# Oil Spill Detection in Terma-Side-Looking Airborne Radar Images Using Image Features and Region Segmentation

**DOI:** 10.3390/s18010151

**Published:** 2018-01-08

**Authors:** Pablo Gil, Beatriz Alacid

**Affiliations:** 1 Department of Physics, Systems Engineering and Signal Theory, University of Alicante, Alicante 03690, Spain; 2 Computer Science Research Institute, University of Alicante, Alicante 03690, Spain; bea.alacid@ua.es

**Keywords:** maritime surveillance, oil spill detection, Side-Looking Airborne Radar, radar detection

## Abstract

This work presents a method for oil-spill detection on Spanish coasts using aerial Side-Looking Airborne Radar (SLAR) images, which are captured using a Terma sensor. The proposed method uses grayscale image processing techniques to identify the dark spots that represent oil slicks on the sea. The approach is based on two steps. First, the noise regions caused by aircraft movements are detected and labeled in order to avoid the detection of false-positives. Second, a segmentation process guided by a map saliency technique is used to detect image regions that represent oil slicks. The results show that the proposed method is an improvement on the previous approaches for this task when employing SLAR images.

## 1. Introduction

Maritime transport and oil rigs are the main causers of marine pollution as a result of tank cleaning and accidents. Ships transport more than 500 million tons of petrol and 300 million of oil products each year and, according to information obtained from the Spanish Maritime Safety Agency (SASEMAR), this maritime traffic spills more than 20 million m^3^/year of both oil and other hydrocarbons into the waters of the European Union.

Oil spill detection is currently carried out by the European Maritime Safety Agency (EMSA), whose CleanSeaNet observation services for oil spill monitoring and vessel detection do so using image from ENVISAT, RASARSAT and SENTINEL satellites, among others. CleanSeaNet is used by governments as a first step toward monitoring oil spills. In a second step, the maritime agencies of each country use aircraft with airborne sensors to monitor the sea surface in more detail. The second step is required because the EMSA satellites can monitor only the same point on the surface of the sea every three days, while and the oil spill continues to expand on the surface, causing significant environmental damage.

Many sensors are used for oil-spill detection, as is presented in [1]. The most common, but not the best, is a radar mounted on satellites and aircraft in order to detect oil on water during searches carried out over a large area. Several efforts have been made to discuss the problem of spill detection, with attention being paid to descriptions of the different satellite radars and oil spill detectability under varying conditions [2,3,4].

There are two ways in which to approach the oil-spill detection problem: studying the characterization of the slick by means of the multi-polarization features of SAR techniques, as occurs in [5,6,7], or using the brightness image obtained from the backscatter signal without considering the parameters of the processes of image acquisition and formation, as in [8,9,10,11,12,13,14,15,16,17,18,19]. Our work falls within the second category. Most of the approaches based on image processing techniques focus on segmenting SAR images in order to identify the dark-spot region that represents the oil slick [8,9,10,11,12], on extracting features so as to recognize the oil slick from previously segmented images [13,14,15], or on classifying dark spots using machine learning techniques [16], statistical classifiers [17,18,19] or/and artificial neural networks [20,21,22,23]. State-of-the-art methods sometimes use a combination of several approaches, as in [24], and on other occasions, the image processing methods are combined with Geographic Information Systems (GIS), as in [25], to provide both the detection and precise location of marine spills. 

Some disadvantages of the methods presented in the state of the art are related to the difficulty involved in automating the oil slick detection task, as mentioned in [26], in which a hybrid system that integrates several automatic and semi-automatic procedures (with human-supervision) is presented. Moreover, the methods are usually tested in an offline process and this may make the performance of the detection task in an onboard detection system difficult, because they are not always designed for input images with sufficient variability (i.e., the acquisition conditions are always chosen in the same way, signifying that the background produced is usually homogeneous or has a specific texture pattern). Another drawback of some of the state-of-the-art methods is the use of cropped images as inputs rather than using images representing a whole mission or full scan (i.e., an image represents a close-up of the oil spill with some background) [9,12,27,28,29]. The input images also frequently show only two types of targets (i.e., the sea and oil slick zones) [30] and when the image shows others, such as land, then pre-processing filters are manually configured to hide or eliminate the pixels in those zones [10,13]. All of these problems still require a solution. Moreover, detection from SLAR images requires finding a solution to these problems, and particularly the last two obstacles, such as working with a full image and using images with other false targets, such as artifacts and noise caused by the flight of the aircraft. Some authors have previously presented a segmentation method based on a boundary [31] or the Markov Random Field (MRF) energy function [32] to reduce or suppress the effect of random noise, low contrast and dayglow contamination.

In particular, the proposed method is based on the segmentation technique category, without considering the multi-polarization features of the radar. But unlike the works in [8,9,10,11,12,13,14,15,16,17,18], the input is a SLAR and not a SAR image. It is very difficult to find works in the state of the art that address oil-spill detection using SLAR images. A recent example in this respect is [22], in which a solution based on a Recurrent Neural Network (RNN) is proposed. The method detects the oil spill using the row of SLAR image as inputs, in which each row represents a scanning of the sea surface in a time t. Other recent works were presented in [23,33]. The authors of [23] present an Auto-encoder Neural Network that uses an unsupervised learning algorithm to segment the oil slick in SLAR images used as input without any previous pre-processing. In [33], two traditional segmentation methods based on graphs and on the concept of J-image are tested for the oil-spill detection task. 

The aim of this work is to present a method based on SLAR image segmentation in order to overcome some of the disadvantages described above. Our method works well with a full image without markers and without cropping the image. In addition, we do not use pre-processing filters to manually eliminate unwanted zones, such as land zones or noise caused by aircraft.

This paper is organized as follows: Section 2 comments on the features of an SLAR image and the difference between the SLAR and the SAR, in addition to describing the dataset obtained from a SLAR mounted on an aircraft. Section 3 describes the proposed method. The experiments showing the behavior of the method are presented in Section 4. Finally, our conclusions and future works are described in Section 5.

## 2. SLAR Image

**Figure 1:**
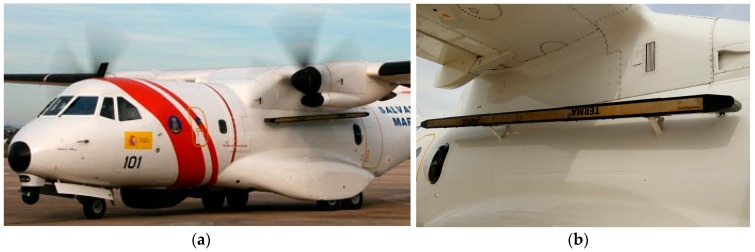
(**a**) Aircraft belonging to Spanish government used to acquire SAR images; (**b**) Detail of SLAR Terma-sensor antenna, which are 12 ft in dimension.

Oil spill detection is usually carried out by governments, principally using two types of sensors: the Synthetic Aperture Radar (SAR), which is installed on satellites, and the Side-Looking Airborne Radar (SLAR), which is installed on aircraft (Figure 1). Unlike other sensors, such as visible spectrum and infrared sensors, both the SAR and the SLAR can monitor a wide area of the sea, even if there is low visibility owing to adverse weather conditions such as clouds or rain. In addition, they can work well at night. The SAR is able to acquire image segments from between 200 and 1400 km, whereas the SLAR image represents image segments with a width and height of 45 km, depending on the number of minutes that the mission lasts. Consequently, SLAR provides images occupying fewer bytes than SAR images. Due to this fact, the use of SLAR enables the aircraft to supply real-time image transmission using a satellite communication link with an operator on earth. Moreover, the acquisition process works on-demand controlled by the flight operator, who commands the aircraft sensors according to flight planning and the type of surveillance mission. 

SLAR images are a visual representation of the interaction of the radar pulse emitted and the surface (sea, land, ship, oil slick, etc.) onto which it is projected. Each pixel in a SLAR image, therefore, represents the backscattering coefficient of that scanned area, and the stronger the backscatter (return signal), the higher the brightness value. 

When both the SAR and the SLAR acquire an intensity image, the oil slicks in it are represented as dark spots (i.e., pixels with low grayscale). This is because dark pixels indicate areas with low backscatter whereas light pixels indicate areas with high backscatter. Nevertheless, it is usual for SLAR images to display other targets, such as ocean phenomena (i.e., the sea surface with an absence of waves owing to little wind) or natural activities (i.e., algae banks and phytoplankton blooms), as dark spots. More specifically, the wind must have a velocity of more than 5 kn. for sea to be sufficiently agitated to produce waves so that the sensor works well. The absence of waves does not allow the SLAR to distinguish between oil slicks and sea zones because the backscatter is low and very similar in both cases.

**Figure 2:**
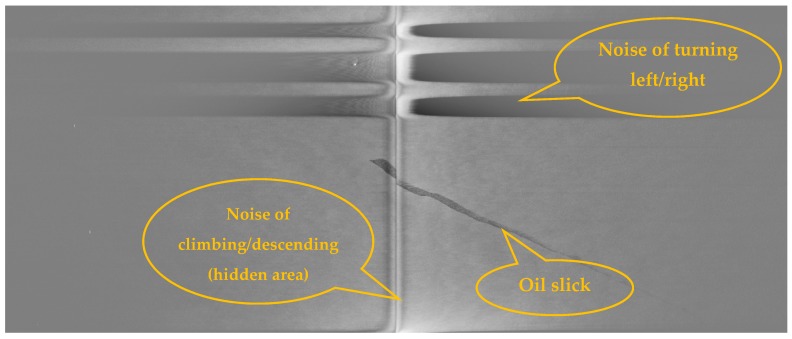
SLAR images acquired by a Terma-sensor mounted on aircraft: Example of oil spill with noise caused by aircraft maneuvers.

Moreover, SLAR images always show other phenomena which do not appear in SAR images and which are represented by dark spots that cause confusion in the oil-slick detection task. The problem is that the location of SLAR antennas, under the aircraft wings (Figure 1b), causes noisy areas in image that may lead to confusion in the detection task. Aircraft maneuvers (turns, slips, etc.) can, similarly, also cause other noisy areas (Figure 2).

Another important difference of SLAR versus SAR is the type of noise. SAR image usually presents granular appearance because of speckle noise, while SLAR presents artifacts. Unlike SAR, SLAR image is actually a mosaic image composed of simultaneous scans performed by each antenna. Therefore, SLAR image can include noise caused by small differences in alignment between both antennas. The position and inclination of each antenna determine the difference between the look angles. This issue influences both the shape and the size of noisy area, named as hidden area in Figure 2. Additionally, this hidden area is modified depending on both the flight altitude and ascent/descent speed. SAR image does not suffer this problem because it does not have any hidden area since it is not a mosaic image.

The width and intensity of noisy areas depend mainly on motion in the longitudinal axis parallel to the fuselage reference line. Due to this rotational movement, the antennas change the look angles. One antenna receives bad measures while the other receives backscatter loss because it looks towards the sky.

Figure 2 shows a SLAR image that represents 7′ and 20″ of a sea surface scan taken by the aircraft shown in Figure 1. This digital image was acquired on a marine surveillance mission on an ascending fight path (between 1552 and 1896 ft.), on which the initial latitude and longitude were N 40°51′ and E 002°22.8′ for the starting point and N 39°54.8′-E 1°11′ for the end point.

The dataset used as input in our experimentation contains 7 SLAR images of oil-slick scenes, which were acquired by a TERMA SLAR 9000 sensor (TERMA A/S, Lystrup, Denmark) mounted on an EADS-CASA CN 235–300 aircraft (EADS CASA/Airbus Group, Madrid, Spain). TERMA SLAR is a Media Wave Radar (MWR) sensor with a frequency of 9.375 MHz that, when mounted on a CN 235–300, can view about 46 km on each side of the aircraft. The beam width of each SLAR antenna is 0.6° horizontal and 19° vertical. 

**Figure 3:**
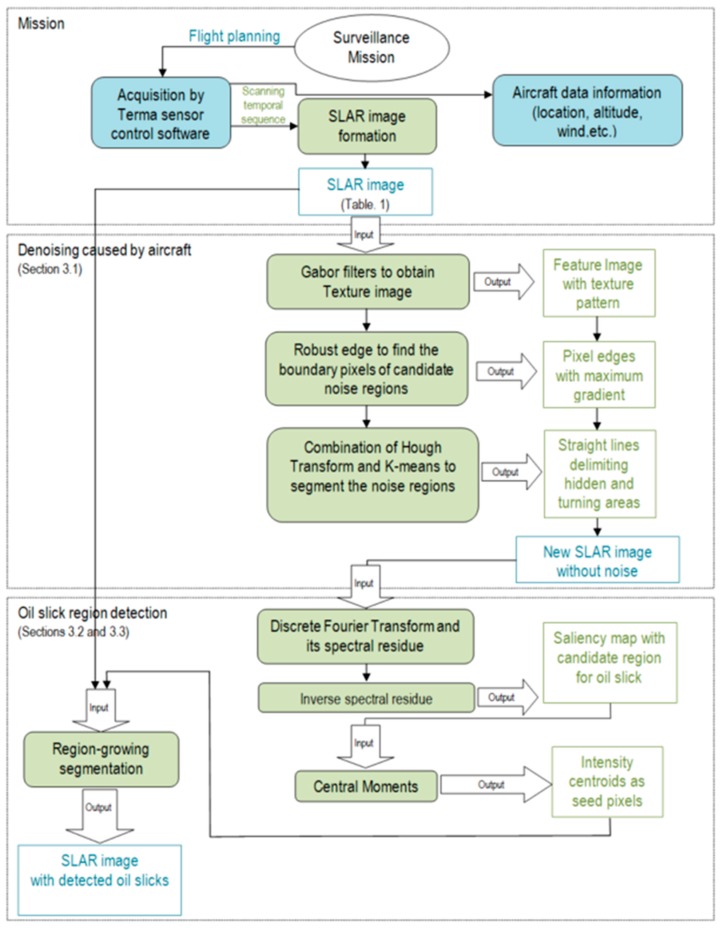
Scheme of method employed to detect oil slicks in SLAR image.

All the images have a resolution of 1157 × 482 pixels and each pixel is 8 bits in depth, according to the digitalization of the Terma sensor control software. They were supplied by the Spanish Maritime Safety and Rescue Agency (SASEMAR). The details of the SLAR images used in the experimentation are described in Table 1. In addition, the proposal method is presented in Figure 3 and some of the used parameters are summarized in Table 2.

## 3. Method

The main contribution of this work is a method with which to detect oil slicks in SLAR images. Figure 3 represents a complete scheme of our method, based on image processing techniques and computer vision. It particularly shows all the processing stages in the detection of oil slicks in SLAR images acquired from an onboard sensor. First, a ‘denoising caused by aircraft’ phase detects the noise caused by aircraft maneuvers while the mission is being carried out, after which an ‘oil slick region detection’ phase detects and locates the oil slicks in the image. The method is discussed in the following sub-sections.

### 3.1. Denoising Caused by the Airplane

SLAR images show noise zones caused by the absence of a radar signal. These noise zones are a consequence of three circumstances: a hidden area under the aircraft fuselage without a radar signal, and changes in location and aircraft turns that take place during an emergency mission and during which the radar signal is lost (Figure 2). The noise zones lead to significant measurement errors which are subsequently registered as erroneous values in the pixels obtained by the digitalized process. It is, therefore, important to be able to automatically detect the pixels caused by noise zones for their subsequent labeling so that they will not be considered in the oil spill detection process. Those pixels that represent noise could cause false positives in the detection process. It is assumed that the hidden area is always unique for each input image, that its location in the image is unknown and that both the number of noise zones and their locations in the image owing to changes and turns are also unknown.

**Figure 4:**
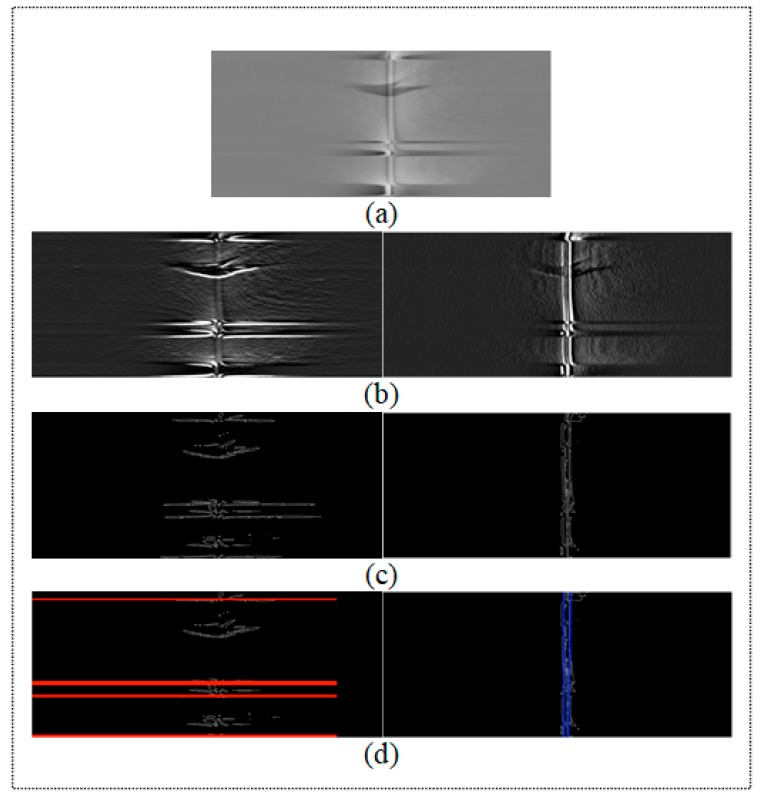
Example of denoising process: (**a**) Original SLAR image; (**b**) Texture pattern obtained by using Gabor filters to detect noise caused by aircraft maneuvers; (**c**) Pixel edges with maximum gradient; (**d**) Straight lines from Hough Transform (k-means has not yet been applied to this point).

The methodology for noise zone detection is implemented as a three-step algorithm. First, the input images I(*x*,*y*) are filtered using Gabor filters [34]; H. Gabor filters use directional masks to detect abrupt changes of intensity in different directions. Second, the Hough transform [35] is applied to the filtered image in order to obtain the straight lines which delimit the abrupt changes as the border of the noise zone. Third, the input image is cropped to eliminate the pixel regions in which the noise zones were detected. A new SLAR image, which is smaller than original image, is consequently built from the input image to be used later in the detection process. Examples of the first and second steps of our algorithm are shown in Figure 4. The detection process carried out using the output of this first phase is described in the following section.

The Gabor filter, H, is a low-pass filter that passes signals with a frequency that is lower than a reference frequency, f. Gabor makes it possible to eliminate texture pattern by detecting the pixels (*x,y*) with intensity values which have a specific level of repetitiveness, i.e., that are homogeneous areas in the image. The Gabor filter is defined as follows:

(1) $$H\left(x,y,\lambda ,\;\theta ,\psi ,\sigma ,\gamma \right)=e^{\left(\frac{{\left(x\cos\theta +y\sin\theta \right)}^{2}+\gamma^{2}-{\left(x\sin\theta +y\cos\theta \right)}^{2}}{2\sigma^{2}}\right)}\cdot e^{i\left(2\pi \frac{x\cos\theta +y\sin\theta }{\lambda }+\psi \right)},$$

where λ defines the wavelength of the sinusoidal factor, θ is the orientation of the normal to the parallel stripes of a Gabor filter,  ψ is the phase offset, σ is the standard deviation of the Gaussian envelope and γ is the spatial aspect ratio. Nevertheless, the output of Terma SLAR sensor is an image digitalized from a time sequence of scanning and H can, therefore, be represented as a discrete filter as follows:

(2) $$H\left(x,y,\;\theta ,f,\sigma \right)=e^{\left(\frac{x^{2}+y^{2}}{2\sigma^{2}}\right)}\cdot e^{i\left(2\pi fx\right)}$$

where  f  represents the frequency that determines the type of texture pattern and θ is its orientation direction. The neighbors considered are controlled with the parameter of the Gaussian standard deviation σ. In our experimentation, we have used θ=92° and σ=5 to obtain good results with our dataset of SLAR images. Note that each turn area is a noisy region between two horizontal imaginary lines whose orientation is close to 0°. Besides, hidden area is another noisy area between two vertical parallel lines whose orientation is close to 90° (Figure 4a). For this reason, we tested values close to 0° and 90° for the texture patterns in a range of ±10°. We got the best results with 0° and 92°. Later, we apply a Gaussian filter $G_{\sigma =5}$ and size 3 × 3 to remove noise due to the response of Gabor filters. We have chosen these values because the effect of filtering with higher values causes too smoothing and consequently, the texture pattern of aircraft maneuvers can be partially erased and this issue difficult the detection of the borders of the noisy areas. A DoG-Canny operator with non-maximum suppression is later used to detect pixels with very different intensity values to those of their neighbors as regards magnitude and gradient direction. In this step, we have used 3 × 3 Gaussian masks and thresholds of 1 and 5 for the Canny method:

(3) $$DoG\left(x,y\right)=\nabla \left[I\left(x,y\right)⊗G_{\sigma }\left(x,y\right)\right]$$

The result of first step provides an image in which the boundary pixels belonging to the noise zones are detected (Figure 4b). We later compute the Hough transform combined with a k-means clustering algorithm to locate straight lines as an approach fitting in order to determine the border of the noise zones. We particularly search for the straight lines with an orientation of 90° and 270° for a hidden area and 0° and 180° for the asymptotes of the parables caused by the aircraft turns (Figure 4a–c). Note that the number of turns and their positions in the image are unknown, and a statistical analysis has, therefore, accordingly been introduced in order to identify those data in the image. What is more, the Hough Transform provides too many straight lines with an identical orientation and close positions, and a k-means clustering algorithm [36] is, therefore, used to cluster straight lines and to show only those lines that define the border in order to divide two turn zones. This analysis is performed by computing the k-means, using different cluster values to search for the k value that minimizes the variance between clusters (Equation (4)). The number of turns in the image is consequently determined by the number of clusters obtained when applying the algorithm. Each turn will, therefore, be delimited by two straight lines, which are determined by the distance between them and the centroid of the turn:

(4) $$\arg\min\overset{k}{\underset{i=1}{\sum }}\underset{\rho_{j}\;\epsilon \;S_{i}}{\sum }{\left||p_{j}-\mu_{i}|\right|}^{2}$$

where each cluster $S_{i}$ is the set of points that defines the straight line separating two turn zones, $\rho_{j}$ is each straight line in the polar coordinates detected by the Hough transform and $\mu_{i}$ is the mean of the points for $S_{i}$.

Once the borders of both the turn and the hidden zones have been calculated in the image, the image is divided into several sub-images using the straight lines of the detected borders as the splitting criterion (Figure 4d). The sub-images representing turns and hidden zones can consequently be eliminated and the method can automatically recompose an entire new image from the merging process using only the remaining sub-images. The new recomposed image will have less noise that could cause false positives in the detection process.

The result of the process carried out to remove the noise caused by the airplane is presented in Figure 4 and the summary of parameters for this step of our method is shown in Table 2.

### 3.2. Candidate Regions Detection to Be Oil Spills

**Figure 5:**
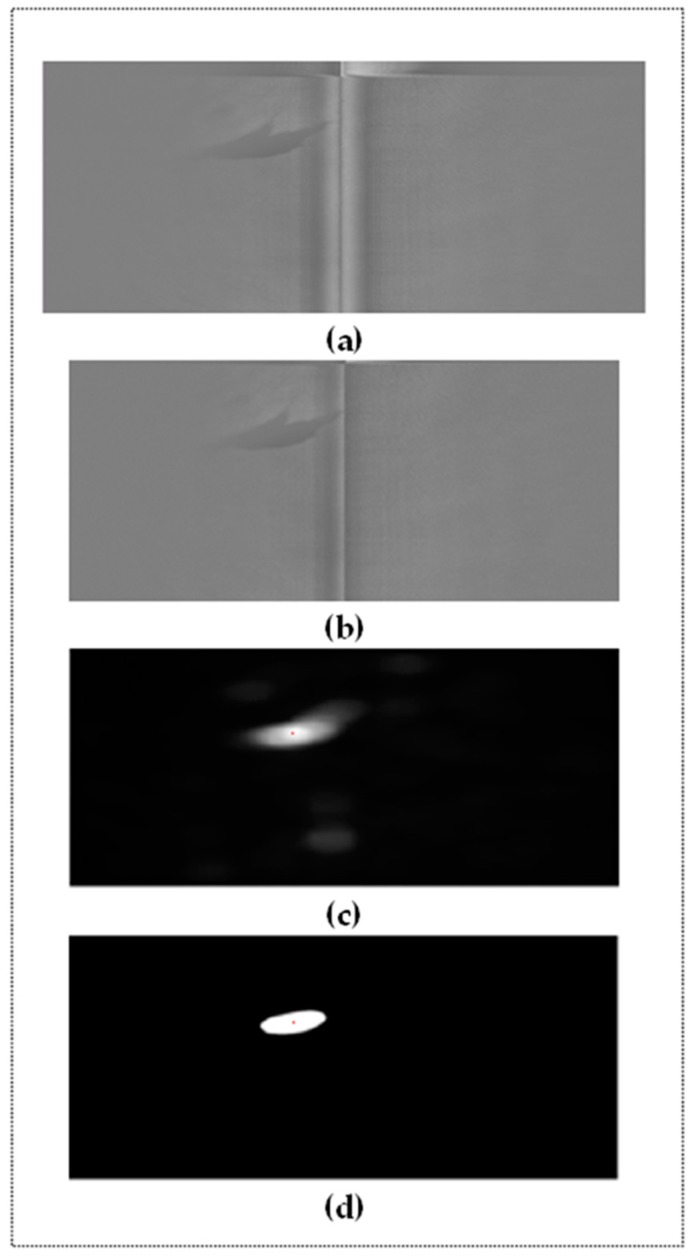
Example of candidate region detection process: (**a**) Original SLAR image; (**b**) New image without noise (i.e., we have applied the process shown in Figure 4); (**c**) Saliency map with spectral residue; (**d**) Thresholding and centroids.

Once the noise has been eliminated, we employ a method based on a Fourier transform and the concept of residual spectral [37]. This method obtains a saliency map that simplifies the representation of the image without noise (Figure 5). The aim is to identify the pixels that provide a greater level of appearance information. The saliency technique is, therefore, used to locate objects from a non-homogeneous and non-uniform background as a previous step to the segmentation of regions method. The location of regions using segmentation methods based on color or grayscale without carrying out preprocessing beforehand does not provide good results when the brightness values of the pixels in the background change. The saliency map, therefore, provides a pre-processing step with which to perform a meaningful segmentation process that detects the pixels representing the oil spills in the image. In order to compute the saliency map, it is first necessary to calculate the spectral residue of the input image (Figure 5c). 

The spectral residue is denoted as R(f), where f=DFT(I(x,y)) is the Discrete Fourier Transform of the image I(x,y). It can also be expressed in polar coordinates in terms of two real functions, amplitude A(f) and phase P(f), where $A\left(f\right)=\sqrt{Re\left\{DFT^{2}\left(I\left(x,y\right)\right)\right\}+Im\left\{DFT^{2}\left(I\left(x,y\right)\right)\right\}}$ is a matrix related to the intensity values of the pixels and P(f) is another matrix with information related to the pixel positions. The spectral residue can, therefore, be denoted using R(f) and is calculated as:

(5) $$R\left(f\right)=\left(\log\left(A\left(f\right)\right)-H_{m}\cdot \log\left(A\left(f\right)\right)\right)$$

where $H_{m}$ is a mean filter that we have chosen with a 3 × 3 sized mask. The map saliency of an image S(I(x,y)) is later calculated using the inverse of the spectral residue generated from the Discrete Fourier Transform (DFT) of image I(x,y) as follows:

(6) $$S\left(I\left(x,y\right)\right)=DFT^{-1}{\left(e^{R\left(f\right)+P\left(f\right)}\right)}^{2}$$

The result of applying the saliency map technique is the generation of several candidate oil-spill regions in the image. Each of the regions detected represents a slick in the SLAR image which could be considered as an oil spill. After obtaining the saliency map, we apply an adaptive thresholding method. We can use any of the methods discussed in [38] to reduce the non-uniformity of intensity in the saliency regions. In particular, Figure 5d shows the result of a binarization using Niblack’s method. We consider a neighborhood window for our seed pixel and then we use as threshold the intensity mean of windows. After, we compare the threshold with the rest of pixels which are not black taking into consideration of standard deviation as tolerance value. 

### 3.3. Reconstruction of Oil Spills 

The detection of candidate oil-spill regions is not sufficient when the purpose is to analyze the changes in the shape and size of the oil slick over time. The SLAR sensors are employed on emergency missions to map, monitor and analyze sea surfaces from aircraft in order to seek oil spills, detect the ships that have caused them and evaluate the disaster according to track the boundary of the oil spill. A reconstruction method must, therefore, be applied to the SLAR image after detecting the candidate regions. The basis of the reconstruction method is a segmentation process that is based on region growing and is used to reconstruct the contour of the oil slick from pixels of candidate regions obtained from the saliency map. The reconstruction, therefore, makes it possible to define the contour pixels of the candidate region and to determine both its size (i.e., using perimeter and area) and shape (i.e., shape descriptor). A comparison of its shape and size at different times is then used to estimate how the oil dissolves in the water, which depends on the oil density, the tides and winds, etc.

The region growing segmentation requires the selection of initial seed pixels to implement the process of pixels clustering. The grayscale value of a seed pixel is used as a reference value to determine the mean homogeneity that the neighboring pixels should have for them to be added to a same cluster. In our work, we use the centroid coordinates of candidate regions obtained from saliency map as seed pixels that will be applied to the original image (SLAR image without any processing). There will consequently be as many seed pixels as the candidate regions that were detected. The centroid is computed by using moments of order up to 1 [39].

In order to perform the process of adding pixels to a cluster, we use an 8-connected neighborhood. Hence, if any adjacent pixel that considers 8-connectivity has a grayscale value similar to the seed pixel, then it is added to the cluster of the seed pixel, and they are classified as new seed pixels for the next iteration of the algorithm. The region growing algorithm follows an iterative process until there are no changes in two successive iterative stages, i.e., the new pixels are not added to grow the region.

When the region growing is applied to the candidate region that is an oil slick (this is a True Positive, TP), the growing algorithm stops because the region is bounded by an edge pixel in which the gradient is high in several directions while other pixels within the region have similar grayscale values because they represent the homogeneous dark spots. On the contrary, the growing algorithm grows throughout the image when the candidate region is not an oil slick (this is a False Positive, FP). The FP is a consequence of imprecisions in the method with which to eliminate the noise zones in a precise manner, shown in Section 3.1.

Finally, our method is able to extract features from the pixels in the candidate regions (both the TP and the FP) in order to determine the physical features of the oil slick for the TP and to ensure that the FP is discarded. It is possible to compute the area (pixels within a dark spot representing the candidate region), perimeter (contour pixels), elongation, bounding box, etc. This matter is not, however, addressed in this work.

## 4. Experiments and Discussion

### 4.1. Results

**Figure 6:**
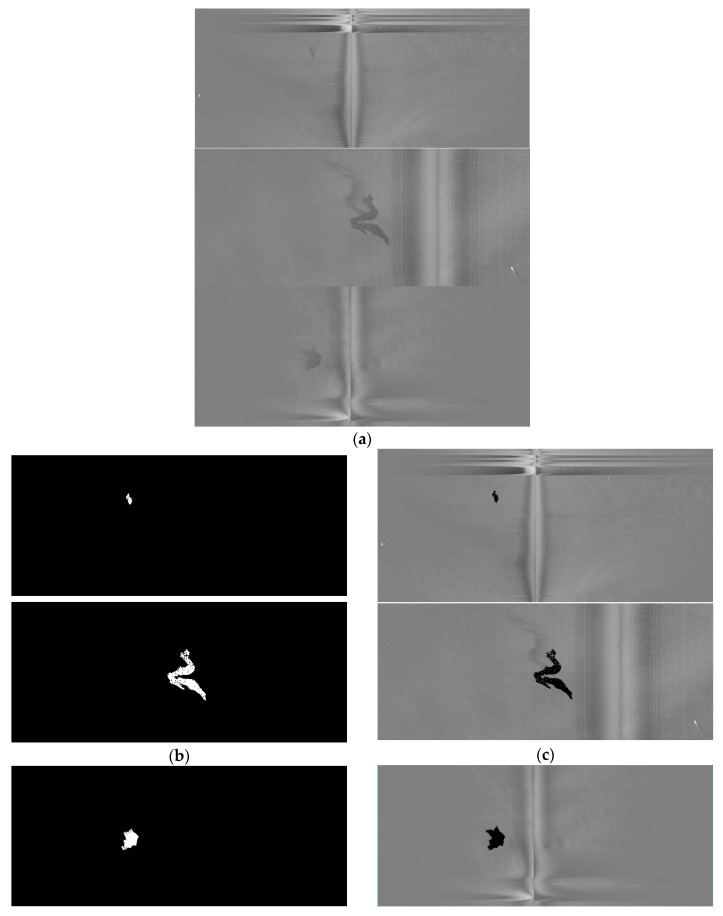
Results of oil-slick detection process with reconstruction: (**a**) Original SLAR image; (**b**) Ground-truth for oil slick area; (**c**) Our oil-slick detection guided by saliency maps and centroids.

This section analyzes the performance of the proposed method (Figure 6). In order to test the reliability of the proposed approach, we have used the test dataset described in Table 1. This dataset represents a set of emergency missions whose purpose is to automatically detect oil slicks using an onboard sensor, such as a SLAR. The feature of the images was described in Section 2.

We applied our approach to several images, always using the same configuration of parameters described in Section 3.1. These values were optimized by tuning the parameters during the experiments until the best visual detection results were achieved. Other parameters are automatically computed in an adaptive manner, signifying that they do not have to be tuned. One example of this is the automatic estimation of seeds from the saliency map, as described in Section 3.2 and Section 3.3. Unlike others, such as the works shown in [9,10,11,12,13,27,28,29,30], the proposed approach always uses full images of the mission as input without using pre-processing masks to eliminate both land and noise pixels. The processing of the full image complicates the detection process but helps achieve a high level of automation. Keeping these issues in mind, we show the capability of our approach as regards detecting different types of oil slicks (i.e., considering the different size, shape, thickness and density of the spill, etc.) in several flight conditions (i.e., varying the wind speed on the sea surface between [0, 27] kn. and with altitudes of between [1061, 4596] ft. In general, all the images were acquired at dusk or at night (Figure 6a) and the results obtained were analyzed by comparing them with a ground truth (Figure 6b). The ground-truth includes all the pixels belonging to the region considered as a true oil slick. The labels of ground-truth pixels are provided to identify our target. Labeled oil slicks are necessary to quantitatively measure the performance when comparing different approaches for segmentation processes.

The ground-truth images are segmented images with human-annotation. The ground-truth was manually done by authors according to help supplied by a flight operator and a marine biologist. To carry out oil-slick annotation, we marked the boundary, pixel by pixel. Later, we filled the area within the boundary using as criteria the visual perception of the human by comparison among the gray value of each interior pixel with both values, mean of boundary and neighboring pixels.

Figure 6c illustrates the results obtained using our approach. As can be seen, a few pixels are considered to be FP. Moreover, the detection method is successful if it is necessary to estimate both the size and shape of oil slicks.

In order to evaluate the performance of the proposed method, we have used the Jaccard index which is a metric widely used for this kind of tasks. It also is known as Intersection over Union (IoU). The Jaccard index *J* is a similarity measure between the segmented image $I_{r}\left(x,y\right)$ (Figure 6a) and the ground-truth $I_{gt}\left(x,y\right)$ (Figure 6b). This way, we compare the performance of our approach to correctly detect oil slicks. The Jaccard index is computed as:

(7) $$J\left(I_{r}\left(x,y\right),I_{gt}\left(x,y\right)\right)=\frac{I_{r}\left(x,y\right)\cap I_{gt}\left(x,y\right)}{I_{r}\left(x,y\right)UI_{gt}\left(x,y\right)}\approx \frac{TP}{TP+FP+FN}$$

The results obtained using Jaccard index are 0.99, 0.87 and 0.76, for the three examples shown in Figure 6. Therefore, the number of pixels detected as TP (number of correctly detected pixels) is much bigger than the sum of FP (number of incorrectly detected pixels) and FN (non-detected or missed pixels) according to the mathematical estimation described in Equation (7).

### 4.2. Comparison with Other SLAR Methods for Oil-Slick Detection

In this section, we compare the proposed method with other published results for oil-slick detection from a SLAR image (Table 3). In particular, we have compared our method with other methods for the detection of oil spills in SLAR images presented in [33], which are based on region segmentation algorithms presented in [40,41]. Unlike the studies shown in [33], which work with sub-images, our method works well with radar full images. On the one hand, our method incorporates the denoising steps to improve the detection process. This additional process improves the success of detection and the results do not, therefore, contain False Positives (FP), nor is their appearance reduced, as occurs with the Graph and JSEG methods.

On the other hand, the proposed method reduces over-segmentation in the detection process. That is, the oil slick is detected as a unique dark spot region and the detected oil spill does not, therefore, appear segmented in several regions as if it were composed of several dark spot regions. All these disadvantages are dealt with by our method, as shown in Table 3.

Table 3 shows two test sub-images containing oil spills and zones which can be look-alikes due to noise caused by aircraft. The first test achieved accuracies of 0.76 versus 0.21 and 0.19, while the second achieved accuracies of 0.73 for this proposed method versus 0.44 and 0.14 for Graph and JSEG method, respectively. The comparison was done using the same evaluation metric again. As can be seen in the table, our approach can be used to discriminate oil spills from image, with a strong background noise, improving the Jaccard index result obtained by other methods.

## 5. Conclusions

In this work, we propose an oil slick detection method for SLAR images using a strategy based on image processing and computer vision techniques without using the characterization of oil slicks obtained from the multi-polarization features of a radar. We use only the intensity image obtained from the backscatter signal without considering radar parameters. The strategy consists of two stages. First, SLAR images are processed to identify noise regions caused by aircraft maneuvers. This step is required to avoid the detection of false positives (i.e., pixels which are not the pixels of an oil slick but have similar brightness features). Second, SLAR images, which have been processed beforehand, are used to detect oil slicks. This process can be performed automatically and can also be carried out by on-boards sensors once the radar scan is digitalized as an SLAR image. 

Although the state of the art almost never contains approaches with which to detect the dark spots of oil slicks in SLAR images, we have made an effort to make a brief comparison between our method and other approaches for the same task using SLAR images as inputs.

As future work, we would like to add deep learning techniques as data augmentation in order to artificially increase the size of the training set and thus improve the detection. Data augmentation consists of transforming the original SLAR image to obtain synthetic samples when the example of missions is not sufficient to design and implement an automatic detection method. In this line, our idea is to also prove the behavior of Convolutional Neural Networks (CNN) in order to simultaneously detect more targets. For example, CNN will provide more flexibility for use in new situations and will make it possible to detect other targets, such as coastlines or ships, at the same time as oil slicks.

## Figures and Tables

**Table 1:** Information of SLAR dataset with final and initial data obtained on missions, respectively.

Image	Location (Latitude-Longitude)	Time Sequence	Wind Speed on Surface Sea	Altitude of Aircraft
Mission 1 (Figure 2)	N 38°19.9′-E 0°23.3′	6 min 39 s	6 kn	1061 ft
	N 38°18.3′-E 0°09.4′		10 kn	1102 ft
Mission 2 (Figure 4)	N 40°04.3′-E 1°28.9′	6 min 24 s	0 kn	1054 ft
	N 40°10.6′-E 1°44.5′		4 kn	1036 ft
Mission 3 (Figure 5)	N 40°09.4′-E 1°21.5′	5 min 49 s	0 kn	4500 ft
	N 40°17.0′-E 1°44.8′		22 kn	4511 ft
Mission 4 (Figure 6)	N 40°51.0′-E 2°22.8′	7 min 20 s	0 kn	1552 ft
	N 40°53.8′-E 2°00.3′		19 kn	1806 ft
Mission 5 (Figure 6)	N 37°36.1′-W 0°29.9′	3 min 12 s	16 kn	4494 ft
	N 37°42.7′-W 0°20.8′		16 kn	4494 ft
Mission 6 (Figure 6)	N 39°53.5′-E 0°48.2′	5 min 29 s	27 kn	4540 ft
	N 39°54.8′-E 1°11.0′		15 kn	4589 ft
Mission 7 (Table 3)	N 39°59.5′-E 1°04.9′	6 min 37 s	24 kn	4596 ft
	N 40°04.0′-E 1°27.8′		6 kn	1172 ft

**Table 2:** Summary of parameters for the proposed method.

Target	Technique an Filters	Values
Detection of abrupt changes of intensity	Gabor Filters (for vertical)	H(21,21,1,0,0,5,1)
	Gabor Filters (for horizontal)	H(21,21,1,92,0,5,1)
Detection of borders for noise areas	Gaussian filter	$G_{\sigma \;=\;1.5}\left(\mathrm{size}\;3\;\times \;3\right)$
	Find edges from Canny detector	(th1 = 1,th2 = 5)
	Hough Transform	H(r = 1, θ *=* 180) H(r = 1, θ = 90)

**Table 3:** Our proposal versus other state-of-the-art methods.

Ground-Truth	Graph Method [33,40]	JSEG Method [33,41]	Proposed Method